# Zieve’s Syndrome: An Under-reported Cause of Anemia in Alcoholics

**DOI:** 10.7759/cureus.4121

**Published:** 2019-02-22

**Authors:** Omar Abughanimeh, Anahat Kaur, Laith Numan, Waled Bahaj, Sheshadri Madhusudhana

**Affiliations:** 1 Internal Medicine, University of Missouri-Kansas City School of Medicine, Kansas City, USA; 2 Hematology and Oncology, University of Missouri-Kansas City School of Medicine, Kansas City, USA

**Keywords:** zieve's syndrome, alcohol, hepatitis, anemia, hemolysis, jaundice

## Abstract

Anemia is a common finding in alcoholics. It is often multifactorial and caused by a combination of liver dysfunction, ineffective erythropoiesis, and poor nutrition. Zieve’s syndrome (ZS) is a clinical syndrome that presents with a triad of jaundice, hemolytic anemia, and hyperlipidemia secondary to alcohol use. Herein, we present a case of a 58-year-old male with a history of liver cirrhosis who presented after a fall due to binge drinking and was found to have severe anemia. Workup was consistent with hemolytic anemia with no source of active bleeding. The patient was managed with supportive treatment and blood transfusions which improved his anemia. However, given his advanced liver disease, he developed encephalopathy and subsequently severe aspiration pneumonia. He died 18 days after admission.

## Introduction

Zieve’s syndrome (ZS) is a triad of jaundice, hemolytic anemia, and hyperlipidemia that develops secondary to alcohol-induced liver injury [1]. It was first described by Dr. Leslie Zieve in 1957. Patients with ZS present with abdominal pain, nausea, and other nonspecific symptoms [1]. Treatment of ZS includes supportive management with blood transfusion and abstinence from alcohol [2]. ZS is an under-reported cause of acute hemolysis in alcoholics [3].

## Case presentation

**Figure 1:**
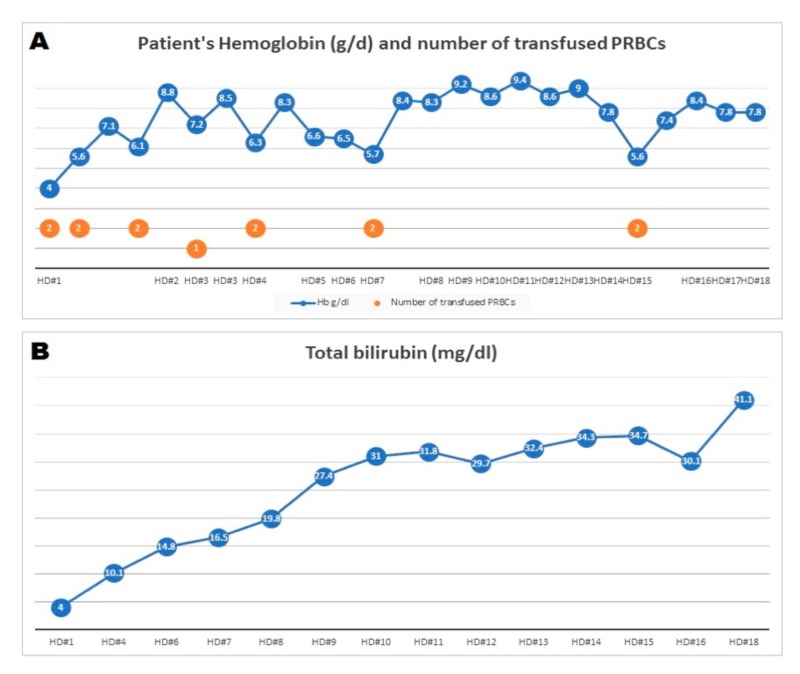
Patient’s hemoglobin level (A) and total bilirubin level (B) during hospital stay PRBC: Packed red blood cells; Hb: Hemoglobin; HD: Hospital day.

A 58-year-old male with a history of liver cirrhosis secondary to alcohol abuse, presented with right hip pain, abdominal pain, and severe anemia. He had been binging on alcohol and sustained a fall prior to his presentation. Computed tomography (CT) scan of the head was negative for intracranial bleed. However, a scan of the abdomen and pelvis showed a fluid collection at the lateral aspect of the right hip concerning for a hematoma. The patient received multiple units of packed red blood cells (RBCs) with no sustained improvement in his hemoglobin (Figure 1A). Esophagogastroduodenoscopy (EGD) showed three columns of non-bleeding grade I varices in the lower third of the esophagus and mild diffuse portal hypertensive gastropathy with no bleeding. A tagged RBC scan was not suggestive of gastrointestinal bleed. CT angiography run-off showed stable muscle and soft tissue hematoma (21 x 6.3 x 5.5 cm) involving the right pelvis and upper leg extending to the level of the knee. A conventional angiography did not show any extravasation from the pelvic and lower limb arteries. Direct and indirect Coombs tests were negative. A blood smear showed macrocytic anemia and thrombocytopenia with schistocytes and acanthocytes. His total bilirubin increased to a maximum of 41 (Figure 1B). Liver Doppler ultrasound showed a heterogenous liver with no focal lesions, patent hepatic and portal veins, and no biliary ductal dilatation. His lipid panel was normal. His initial laboratory workup, along with his labs on day seven which showed evidence of hemolysis, is shown in Table 1. The patient was diagnosed with atypical ZS and supportive treatment was recommended. Unfortunately, his hospitalization was complicated by encephalopathy, aspiration pneumonia, and septic shock. He died due to his comorbidities.

**Table 1:** Lab results from hospital day 1 and day 7 INR: International normalized ratio; AST: Aspartate aminotransferase; ALT: Alanine aminotransferase; LDL: Low-density lipoprotein; HDL: High-density lipoprotein.

Labs/Hospital day	Hospital day 1	Hospital day 7
Complete blood count		
Hemoglobin (g/dl)	4-7.1	5.7-8.4
Platelet count (per cmm)	58,000	94,000
Liver function test		
INR	1.6	1.5
Total bilirubin (mg/dl)	4	16.5
AST (U/L)	117	82
ALT (U/L)	21	22
Lipid panel		
LDL (mg/dl)	Not done	86
HDL (mg/dl)	Not done	26
Total cholesterol	Not done	130
Triglycerides (mg/dl)	Not done	92
Hemolytic workup		
Coomb’s test (Direct & indirect)	Not done	Negative
Reticulocyte count	Not done	16.2 %
Haptoglobin (mg/dl)	Not done	10
Lactate dehydrogenase (U/L)	Not done	505

## Discussion

ZS is a clinical syndrome that manifests as a triad of jaundice, hemolytic anemia and hyperlipidemia due to alcohol-induced liver injury. It was first described by Dr. Zieve in 1958 when he studied 20 patients with hemolytic anemia associated with alcohol abuse [1]. Studies confirm that ZS is under-reported and is often undiagnosed or misdiagnosed [3]. Liu et al. performed a comprehensive search on the PubMed database from 1958-2017 which revealed 120 reported cases, mostly in non-English literature (96 papers) [4]. The incidence of ZS is estimated to be one in 1,600 admissions [5].

Three classical features of ZS are jaundice, hyperlipidemia, and hemolytic anemia. Jaundice and hyperbilirubinemia result from hemolysis and cholestasis due to alcohol-induced liver injury [4]. Hyperbilirubinemia in ZS is usually severe as seen in our patient with maximum bilirubin level up to 41. This occurs as a result of several contributing factors including hepatocellular injury, hemolysis and spur cell anemia [6]. Current alcoholic hepatitis scoring systems, therefore, overestimate morbidity and mortality from liver disease based on abnormally high total bilirubin level without considering other underlying factors [7]. Hyperlipidemia, however, is usually transient and can be missed after 1-2 weeks of initial insult [1,8]. Thus, atypical presentation of ZS can occur with a normal lipid panel. In Dr. Zieve’s study, the incidence of hyperlipidemia was 50%, this was attributed to delay in testing the lipid panel and advanced liver dysfunction which impaired lipid production [1]. This atypical presentation was seen in our case where lipid panel checked on day seven of hospital stay was within normal limits.

The pathophysiology of hemolysis in ZS is not fully understood. Dr. Zieve suggested that hyperlipidemia and presence of abnormal lipids (possibly lysolecithin) plays a role in hemolysis by disrupting RBC membrane [1]. In 1968, Balcerzak et al. evaluated six patients with ZS, the study showed that both autologous and transfused healthy donor RBCs were hemolyzed in patients with ZS [9]. This led to the conclusion that an extracorporeal abnormality did not explain the entire process. Later, studies showed that vitamin E deficiency secondary to alcohol use can result in pyruvate kinase instability which affects RBC metabolism leading to hemolysis [10-11].

It is important to know that ZS can present with other symptoms in addition to the triad. For example, abdominal pain was present in all 20 cases reported by Dr. Zieve. Other nonspecific symptoms include nausea, vomiting, malaise, weakness, and low-grade fever [1]. ZS can mimic alcoholic hepatitis as the two conditions share findings such as alcohol use, jaundice, and anemia [3,7]. A good way to differentiate between them is to determine the type of the anemia; ZS causes hemolytic anemia while alcoholic hepatitis causes macrocytic anemia. Delayed diagnosis of ZS is not uncommon [12] and this is one of the challenges we faced in the management of our patient.

Treatment of ZS is mainly supportive with blood transfusions and encouraging abstinence from alcohol [2]. Liver transplantation has been suggested as a treatment if there is no improvement in liver function and hemoglobin despite alcohol abstinence and the patient continues to remain transfusion dependent [13]. Generally, jaundice, anemia, and hyperlipidemia resolve within a few weeks of the illness [1].

## Conclusions

Even though ZS is rarely reported, it should be suspected in patients with worsening hemolytic anemia with no apparent explanation, especially in alcoholics. Being aware of ZS can limit workup, cost and help avoid using unnecessary drugs that can worsen the condition.
